# The Italian Pediatric Network

**DOI:** 10.1186/1824-7288-40-S1-A38

**Published:** 2014-08-11

**Authors:** Riccardo Longhi, Maria T Ortisi, Gianluca Gargantini, Domenico Minasi, Nicoletta Matera, Luciana Parola

**Affiliations:** 1 U.O di Pediatria, Ospedale Sant'Anna, S.Fermo della Battaglia,Como, 22020, Italy; 2 U.O di Pediatria e Neonatologia,Ospedale di Lodi,Lodi, 26900, Italy; 3 U.O di Pediatria,Ospedale Santa Maria degli Ungheresi, Polistena,18061, Italy; 4 U.O di Pediatria e Neonatologia,Ospedale Fornaroli, Az.Osp.Ospedale civile di Legnano,Magenta, 20013, Italy

## 

The Italian Pediatric Network is now 8 years old.

The number of pediatric Departments has increased from the 19 that in 2006 began a feasibility study of the project, to the 129 of today.

The objectives of the network are: scientific research, surveillance of rare events, monitoring of complex phenomena, standardization of diagnostic criteria and therapeutic processes, creation of a hospital network capable of allowing a fast and profitable exchange of informations, evaluation of the degree of implementation of clinical Guide Lines.

The Network was created by the Working Group for the Accreditation and Quality Improvement (GSAQ) of the Italian Society of Pediatrics Clinical. Date are recorded at the time of discharge by a single operator for each hospital and loaded into anonymous electronic case report forms, different for each pathology, prepared with the help of subspecialty Scientific Societies and available in the Pediatric Network website [http://networkpediatrico.sip.it].

It is not possible to report here all the results obtained from the analysis of the data concerning the first four pathologies investigated (idiopathic trombocytopenic purpura, diabetes at onset, bacterial meningitis after the neonatal period, acute asthma after the second year of life).

We will present just a few data on the fifth pathology: acute gastroenteritis in children below 5 years of age. 31 centers filled 612 case report forms in 7 months.

It is interesting to compare the diagnostic and therapeutic policies of the partecipating units with the indications of the ESPGHAN Guide Lines. The appropriateness of admissions was rather low, beeing only 42,5% altogether, and minimal for patients admitted under pressure from the family, 2%. Appropriateness of treatments: only 2/3 of the patiens have been correctly treated showing complete or almost complete adherence to the recommendations (no more than 2 major violations or one major and two minor). Major violations were considered those that might negatively affect the course of the disease, unnecessarely increase the cost of treatment or any violation to high grade recommendations, minor violations those that did not change the course of the disease, even if not appropriate and all interventions in contrast with low grade recommendations.

The most frequent violations were: microbiological studies, wrong diet prescriptions, use of unrecommended antidiarrhoeal drugs or of probiotics lacking evidence of efficacy, prescription of antibiotics.

Two other report forms, concerning bronchiolitis and ALTE, will be officialy presented at this meeting and are ready to be launched in the network.

